# Case Report: A novel *de novo* heterozygous truncating mutation in *MED12L* identified in a Chinese autistic boy

**DOI:** 10.3389/fpsyt.2026.1824663

**Published:** 2026-07-13

**Authors:** Zhiliu Wu, Chuanyong Xu, Jierong Chen, Zhen Wei

**Affiliations:** 1 Shenzhen Maternity and Child Healthcare Hospital, Women and Children’s Medical Center, Southern Medical University, Shenzhen, Guangdong, China; 2 School of Psychology, Shenzhen University, Shenzhen, Guangdong, China

**Keywords:** autism spectrum disorder, case report, kinase module, MED12L, truncating mutations

## Abstract

**Background:**

Autism spectrum disorder (ASD) is a highly heterogeneous neurodevelopmental disorder. A previous study by Nizon et al. indicated that some children with intellectual disability (ID) carrying *de novo MED12L* mutations exhibited mild to moderate autistic features. However, the relationship between *MED12L* and ASD remains unclear.

**Case presentation:**

Here we reported a male child with severe autistic features carrying a novel *de novo* heterozygous truncating mutation of *MED12L* (NM_053002.5:c.586C>T, p.(Arg196Ter)). He was diagnosed with ASD according to ICD-11 and DSM-5 criteria. Clinical examination indicated that this child exhibited severe autistic features and several dysmorphic features, including a flat nasal bridge, bulbous nasal tip, thin upper lip, and triangular face. Magnetic resonance imaging (MRI) of the brain revealed an enlarged perivascular space in the right temporal lobe.

**Conclusion:**

This case demonstrates that this *de novo* heterozygous truncating mutation in *MED12L* may be involved in the development of ASD, and haploinsufficiency of MED12L may be associated with severe autistic features. Obvious clinical manifestations and dysmorphic features in this child with a truncating mutation in *MED12L* expand the phenotypic spectrum of MED12L-related cases and warrant further functional studies to elucidate the relationship between MED12L and ASD.

## Introduction

Autism spectrum disorder (ASD) is a neurodevelopmental disorder characterized by difficulties in social interaction and repetitive behaviors (1). Family and twin studies have found that genetic factors play a major role in the development of ASD (2, 3). Previous whole-exome sequencing studies have identified multiple rare *de novo* mutations that contribute to the development of ASD. A whole-exome sequencing study by Sanders et al. found that the rate of non-synonymous *de novo* variants in ASD probands was higher than that in sibling controls. Meanwhile, nonsense *de novo* variants showed significant enrichment in probands compared to unaffected siblings (4). Results from a whole-exome sequencing study in 2,500 simplex families revealed that *de novo* coding mutations contribute to approximately 30% of all simplex ASD diagnoses. In addition, *de novo* likely gene-disrupting (LGD) mutations are enriched for chromatin modifiers, FMRP-associated genes, and embryonically expressed genes (5). Therefore, these findings indicate that *de novo* LGD mutations may be involved in the development of ASD.

Previous genetic studies have identified genes encoding the subunits of the kinase module, such as *MED13* and *MED13L*, as high confidence genes associated with ASD. Two large-scale exome sequencing studies found that *de novo* and loss-of-function variants in *MED13* and *MED13L* might increase the likelihood of ASD (5, 6). By utilizing chromosomal microarray analysis and exome sequencing, several family studies have also found that individuals carrying *de novo* microdeletions, nonsense variants, frameshift variants, or missense variants of exons in *MED13* and *MED13L* were diagnosed with autism or manifested intellectual disability (ID) with autistic features (7, 8). In addition, hemizygous missense mutations in *MED12* have been found in individuals with several X-linked neurodevelopmental disorders, such as Opitz–Kaveggia syndrome, Lujan syndrome, and Ohdo syndrome. These MED12-related genetic syndromes are characterized by ID and several typical facial or dysmorphic features. Moreover, a previous study indicated that some individuals carrying *de novo* exonic variants in *MED12* also exhibited autistic features (9). Consequently, these findings show that *de novo* mutations in genes encoding the subunits of the kinase module are associated with ASD.

Mediator complex subunit 12 like gene (*MED12L*) [OMIM 611318], another gene encoding the subunit of the kinase module, is located on chromosome 3q25. Previous linkage analyses revealed that chromosome 3q25-q27 was a candidate susceptibility locus associated with ASD (10). A genome-wide association study in families also found that three single-nucleotide polymorphisms (rs7649494, rs2872090, and rs10935907) in chromosome 3q25.22 were nominally associated with ASD (11). Remarkably, a study by Nizon et al. revealed that children with ID carrying *de novo* copy number variants and a frameshift mutation in *MED12L* exhibited mild to moderate autistic features (12). Several case reports from different countries have also found some individuals carrying nonsense mutations and deletions in *MED12L* exhibit autistic features (13, 14). However, the relationship between *MED12L* and ASD needs to be further clarified by analyzing the clinical features of individuals carrying different types of *de novo* mutations in *MED12L*.

In this case report, a novel heterozygous *de novo* truncating mutation in *MED12L* was found in a male autistic child. Compared to the cases reported in previous studies, we observed that *de novo* truncating mutations that resulted in haploinsufficiency of MED12L might be associated with severe autistic features. This child also manifested several dysmorphic features and physical conditions observed in other individuals carrying *MED12L* truncating mutations. Magnetic resonance imaging (MRI) of the brain revealed an enlarged perivascular space (PVS) in the right temporal lobe.

## Case presentation

This child was a boy (aged 6 years and 9 months) with a birth weight of 3.0 kg and a birth length of 49 cm. At 40 weeks, the boy was born as the first child of his 32-year-old mother via normal delivery. His mother denied a history of neonatal hypoxia, asphyxia, and other diseases. No positive family history was found in three generations of both maternal and paternal lines. His mother reported that the child had chronic constipation.

This child was referred to the Child and Adolescent Psychiatry Clinic in Shenzhen Maternity and Child Healthcare Hospital at the age of 17 months because of poor social communication and speech delay. This child experienced developmental delays after birth. He did not speak simple words, such as “ma” and “ba”, until 16 months and did not walk until 17 months. When he needed help, he directly grabbed his parents’ hands to get objects instead of pointing at objects with his fingers. Simultaneously, he exhibited some obvious autistic features, including poor eye contact, no response to his name, and restricted interests. At his age of 17 months, the Gesell Development Schedules showed significant delays in personal–social and language, as well as adaptive behaviors and fine motor skills (Development Quotient (DQ): personal–social, 59; language, 47; adaptive behaviors, 65; fine motor, 70; and gross motor, 76) (15). The Childhood Autism Rating Scale (CARS) score was 33.5. The total score of the Autism Diagnostic Observation Schedule (ADOS) was 19, with seven points for communication and twelve points for social interaction.

When examined at the age of 2 years and 4 months, he only spoke two words (“no” and “mama”) and understood several one-step verbal commands such as “sit” (“Zuo Xia” in Chinese) and “throw away” (“Diu Diao” in Chinese). In addition, this child was addicted to spinning objects and observing digital alphabet images. At this age, the Gesell Development Schedules still showed significant delays in all five subscales (DQ: personal–social, 38; language, 39; adaptive behaviors, 58; fine motor, 61; and gross motor, 68). The CARS score was increased to 41. The total ADOS score remained 19. Meanwhile, wakefulness and sleep electroencephalogram (EEG) at this age did not show abnormal signals.

At the age of 4 years and 8 months, he could speak several simple sentences, including two or three words, and exhibited poor social interaction with peer children. He preferred to observe the metro map and piano score alone. Moreover, the Griffiths Development Scale (Chinese edition) showed severe delays in the subscales of locomotor, personal–social, language, eye–hand coordination, visual–spatial performance, and practical reasoning (16). The percentile of the general quotient of all six subscales was below 2.5%. The CARS score was 38.5, and the total score of ADOS was 12, with five points for communication and seven points for social interaction. According to ICD-11 and DSM-5 criteria, the clinical features of this child were consistent with autism spectrum disorder. Through physical examination, several dysmorphic features, including flat nasal bridge, bulbous nasal tip, thin upper lip, and triangular face, were identified in this child (**Figure 1**). The head circumference was 51 cm, and the testicular volume was 2 mL. The height and weight were 107 cm and 16 kg, respectively. MRI of the brain revealed an enlarged PVS in the right temporal lobe. No obvious structural abnormalities were detected in the brain (**Figure 2**).

**Figure 1:**
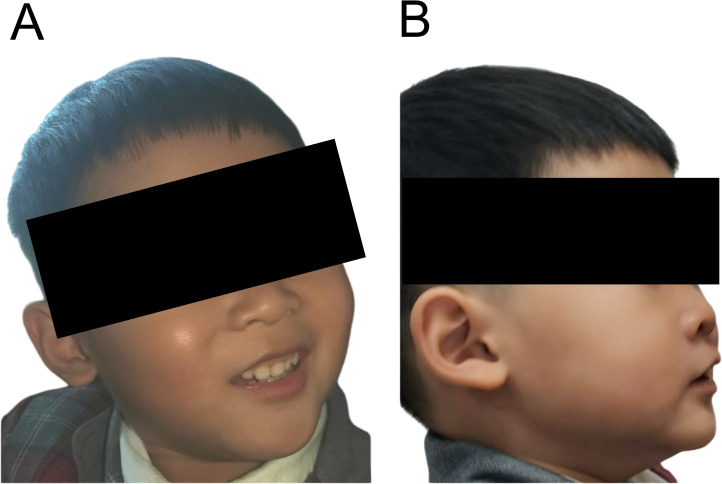
Dysmorphic features of this autistic child. **(A)** Triangular face. **(B)** Bulbous nasal tip and thin upper lip.

**Figure 2:**
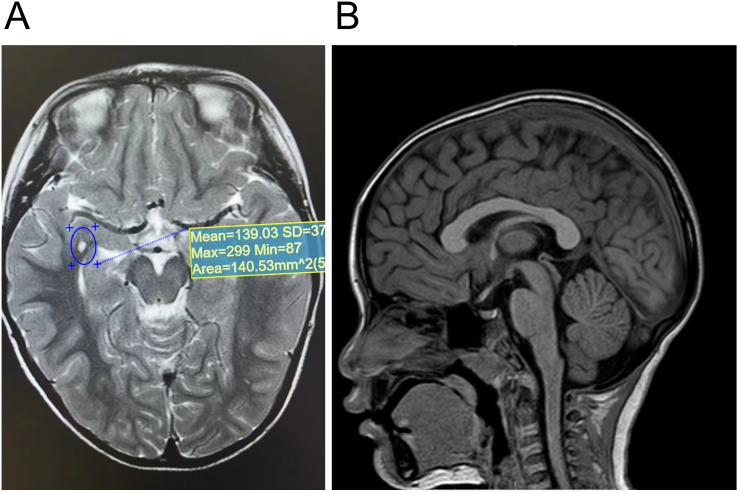
Axial and sagittal slices of this child’s brain acquired on an MRI. **(A)** An enlarged perivascular space in the right temporal lobe was observed on T2 weighted imaging. **(B)** No hypoplasia or atrophy of the corpus callosum was observed on T1 weighted imaging.

Additionally, a test of the Wechsler Preschool and Primary Scale of Intelligence—Fourth Edition was performed at the age of 6 years and 9 months. The test showed that his intelligence quotient of the full scale was 82, with low verbal comprehension score and high visual–spatial and working memory scores (verbal comprehension: 66, visual–spatial: 121, fluid reasoning: 77, working memory: 100, and processing speed: 77). Meanwhile, his attention and response control quotient were measured using the Integrated Visual and Auditory Continuous Performance Test (IVA-CPT). The auditory and visual stimulation parameters applied in the test were provided by the American Braintrain company. His response control quotient (RCQ) of the full scale was 138, including auditory RCQ 137 and visual RCQ 134. The attention quotient (AQ) of the full scale was 140, including auditory AQ 140 and visual AQ 130. His hyperactivity quotient was 108.

Based on the information provided by his parents, this child received continuous intervention from social resources before starting elementary education. His parents reported no obvious improvement in his ability to interact socially and communicate. A timeline with relevant data from the development assessment and detailed information about the intervention is shown in **Supplementary Figure 1**.

### Discovery and validation of genetic mutation

We performed whole-exome sequencing to explore rare exome mutations or copy number variants in this family. DNA was isolated from peripheral blood using a DNA Isolation Kit (Blood DNA Kit V2, CW2553). DNA libraries were prepared using the KAPA Library Preparation Kit (Kapa Biosystems, KR0453) following the manufacturer’s instructions. DNA libraries were sequenced on the Illumina NovaSeq platform as paired-end 200-bp reads. Primary data were generated using Illumina Pipeline (version 1.3.4). Low-quality reads were excluded. The quality control criteria included an average depth of >100× and 20× coverage of >98%. The Burrows–Wheeler Aligner (BWA) was employed to align the clean reads to the human reference genome from the NCBI database (build GRCh37). Variant calling was performed using the Genome Analysis Toolkit (GATK). Variants with a minor allele frequency (MAF) <0.05 in public population databases were included. Pathogenic variants were evaluated using the dbSNP, OMIM, HGMD, and ClinVar databases. Eventually, a *de novo* heterozygous truncating mutation in exon 5 of *MED12L* (NM_053002.5:c.586C>T, p.(Arg196Ter)) was detected in this child (**Figure 3A**). Primers 5′- TTGGGTGGGTATCCTTTTCTGA-3′ and 5′- AGGAATTTGCTGTCTTCTTAAAAGCA-3′ were used to amplify the exonic region spanning this truncating mutation in *MED12L*. This truncating mutation was confirmed by Sanger sequencing, and co-segregation analyses among the family were also conducted (**Figure 3B**). No copy number variants (above 100 kb) of the exome were detected in this family. A diagram of the position of this *de novo* heterozygous truncating mutation in *MED12L* was drawn using IBS 2.0 Web Server (17).

**Figure 3:**
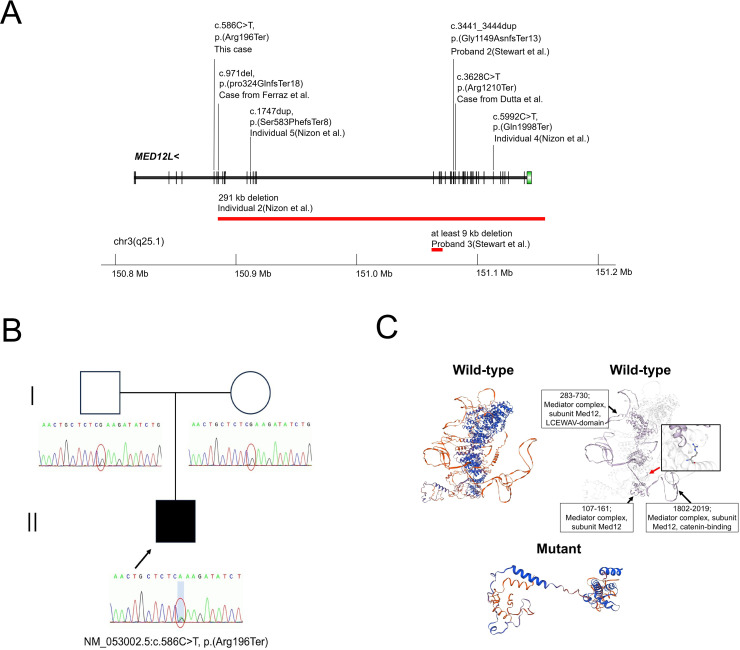
Mutation analysis of this novel *de novo* truncating mutation in MED12L. **(A)** Position of this *de novo* heterozygous truncating mutation of *MED12L* (rare truncating mutations found by Nizon et al. were also noted in this figure; red represents deletion). **(B)** Pedigree of the proband with this *de novo* heterozygous truncating mutation of *MED12L*. (An arrow indicates the proband.) Sequence data for mutant alleles and their family segregation are shown below the symbol. 3D homology modeling. The 3D homology model was generated based on the available MED12L model in the SWISS-MODEL repository (https://swissmodel.expasy.org/) for the wild-type and mutant forms. Black arrows indicate the protein domains of MED12L. The red arrow indicates arginine.

### Annotation and function analysis of variants

Based on the Human Gene Mutation Database at the Institute of Medical Genetics in Cardiff (HGMD, https://www.hgmd.cf.ac.uk/), the 1000 Genomes Project, and gnomAD, we identified known disease-causing mutations. According to the classification guidelines of the American College of Medical Genetics and Genomics, phenotype-associated variants predicted to be pathogenic, likely pathogenic, and of uncertain significance were selected (18). Additionally, the Mutation Taster 3 and CADD scores were used to predict the pathogenicity of the detected mutations (19, 20).

According to the classification guidelines of the American College of Medical Genetics and Genomics, the pathogenicity of the *de novo* truncating mutation in *MED12L* was classified as likely pathogenic. The CADD score for this mutation was 36. Mutation Taster 3 classified this mutation as disease-causing. In the general population, the gnomAD database showed that the frequency of predicted loss-of-function variants (pLoFs) of *MED12L* was 0.00095 (60/63046) and the probability of being loss-of-function intolerant (pLI) of *MED12L* was 1.000. Using the SWISS-MODEL repository (21), we found that this truncating mutation resulted in the elimination of two protein domains, including eukaryotic Mediator 12 subunit domain (Med12-LCEWAV) and eukaryotic Mediator 12 catenin-binding domain (Med12-PQL) (**Figure 3C**).

## Discussion

Through whole-exome sequencing, we found that this autistic child carried a *de novo* heterozygous truncating mutation in *MED12L* (NM_053002.5: c.586C>T, p.(Arg196Ter)) that was not previously reported (12).

In the study of Nizon et al., individuals carrying different types of rare exonic variants in *MED12L* exhibited various clinical features. Three male patients carrying copy number variants and one male patient carrying a frameshift mutation that resulted in protein truncation exhibited mild to moderate autistic features. The autistic features in a male patient who carried a 291-kb deletion starting from exon 6 of *MED12L* were more serious than those in the other three male patients who carried two duplications and a frameshift mutation in exon 12 of *MED12L* (12) (**Figure 3A**). Remarkably, in this case, the male child carrying a *de novo* truncating mutation in exon 5 of *MED12L* exhibited severe autistic features. By comparing the clinical features of the individuals with truncating mutations involving *MED12L* from the study of Nizon et al. and this case, we found that individuals carrying *MED12L* truncating mutations that eliminated more exons manifested more serious autistic features (**Table 1**). However, in this case report, the clinical phenotypes of this boy differed from those reported by Nizon et al—for example, at this child’s age of 6 years and 9 months, a test of the Wechsler Preschool and Primary Scale of Intelligence showed that his intelligence quotient of the full scale was 82 (below average), not meeting the ICD-11/DSM-5 criteria for intellectual disability (quotient cutoff ≤70 with adaptive deficits). Other case reports have also found that some individuals carrying truncating mutations and deletions in *MED12L* exhibited autistic features (13, 14, 22), but the severity of autistic features was not evaluated (**Table 1**). These findings suggest that protein-truncating mutations in *MED12L* might be associated with autistic features, and haploinsufficiency of MED12L might increase the likelihood of severe autistic features.

**Table 1:** Comparison of the clinical features of individuals with truncating mutations involving MED12L from other studies and this case.

Individual	This case	Individual 2 (study of Nizon et al.)	Individual 4 (study of Nizon et al.)	Individual 5 (study of Nizon et al.)	Case from Ferraz et al.	Case from Dutta et al.	Proband 2 (study of Stewart et al.)	Proband 3 (study of Stewart et al.)
Truncating mutation in *MED12L* (according to NCBI reference sequence NM_053002.5, NC_000003.11)	g.150873977 C>T, c.586C>T, p.(Arg196Ter)		g.151129252C>T, c.5992C>T, p.(Gln1998Ter)	g.150906260dup, c.1747dup, p.(Ser583PhefsTer8)	c.971del, p.(pro324GlnfsTer18)	c.3628C>T, p.(Arg1210Ter)	c.3441_3444dup p.(G1149NfsTer13)	
Size of CNV (Mb)		291 kb deletion						del(3)(q25.1q25.1) at least 9 kb
Proximal breakpoint (Hg19)		150,876,508						NA
Distal breakpoint (Hg19)		151,167,962						151,075,210
Inheritance	*de novo*	*de novo*	NA	*de novo*	NA	Maternal	NA	NA
Origin	China	France	Ukraine	USA	Brazil	USA	USA	USA
Gender	Male	Male	Female	Male	Male	Female	Male	Male
Birth term (WG)	40	At term	NA	37	NA	40	39	39
Birth length (cm)	49	53	NA	48.5	NA	48.3	47	NA
Birth weight (grams)	3,000	4,000	NA	3,000	NA	3,260	3,005	2,920
Age at ASD diagnoses	4 years 8 months	NA	NA	NA	NA	2.5 years	4 years	NA
Neurological abnormalities								
Intellectual disability	Below average (IQ 82 at 6 years 9 months)	Moderate	Moderate	Mild	NA	+	NA	+
Hypotonia	–	–	NA	+	NA	NA	NA	–
Motor delay	+ (walking at 17 months)	–	NA	+	+	+	NA	+
Speech impairment	+ (WISC-IV verbal comprehension score: 66)	+	+	+	+	+	+ (with severe articulation phonological disorder)	+
Autistic features	++	++	–	+	NA	Present (uncertain severity)	Present (uncertain severity)	Present (uncertain severity)
Attention deficit	–	+	–	+	NA	+	+	+
Hyperactivity	–	–	–	+	NA	+	+	+
Seizures	–	–	–	–	NA	NA	–	NA
Abnormal EEG	–	–	–	–	NA	NA	–	NA
Abnormal brain magnetic resonance imaging	An enlarged perivascular space of the right temporal lobe	NA	Agenesis of the corpus callosum, enlargement of the posterior aspect of the right and left lateral ventricle	Mildly hypoplastic corpus callosum	NA	NA	–	NA
Extra-neurological abnormalities								
Gastrointestinal anomalies	Chronic constipation	Gastroesophageal reflux	–	Feeding difficulties in early infancy, moderate chronic constipation	NA	Constipation, Incontinence, Loose stool	NA	Constipation
Congenital malformations	–	–	–	Suspected VSD prenatally but normal echocardiogram at birth, hypospadias, voiding dysfunction	NA	NA	–	NA
Skeletal abnormalities	–	–	Very large knees, appears to have bony prominence medially	–	NA	–	advanced bone age	NA
Hands and feet anomalies	–	Long appearing fingers	Fingers-fetal padding, 5th hypoplastic nails	–	NA	–	NA	NA
Sensory abnormalities	–	–	–	–	NA	NA	NA	NA
Sleep disturbance	–	NA	NA	NA	NA	+	+ (obstructive sleep apnea and frequent night awakenings)	–
Dysmorphic features								
High forehead	–	–	–	+	NA	NA	–	–
Downslanted palpebral fissures	–	–	–	–	NA	NA	–	–
Fullness of the upper eyelids	–	+	–	–	NA	NA	–	–
Prominent nasal bridge	–	+	+	–	NA	NA	–	–
Flat nasal bridge	+	–	–	–	NA	NA	–	–
Bulbous nasal tip	+	–	–	+	NA	NA	–	–
Open mouth	–	–	–	–	NA	NA	–	–
High, narrow palate	–	–	+	–	NA	NA	–	–
thin upper lip	+	–	–	+	NA	NA	–	–
triangular face	+	–	–	+	NA	NA	–	–
oligodontia	–	–	–	–	+	NA	–	–
Additional genetic findings								
	–	NA	NA	arr[hg19]4q34.3 (178,557,799–179,142,775)×3 (non-disease-associated region)	NA	c.327G>A, p.(Lys109=), in *GAMT*; c.542C>A, p.(Ala181Glu), in *TNFRSF13B*	NA	c.1168C>T, p.(R390Ter), in *TRMT1*
Karyotype	NA	Normal	NA	46,XY,t(9,18)(p13,q12.2)	46,XY,t(1;2)(p33;p22), t(5;9)(p15;q21)	Normal	NA	NA

Autistic features are classified as mild to moderate (+) or moderate to severe (++). Other signs are considered as present (+) or absent (−). NA indicates not available.

Additionally, some dysmorphic features including a bulbous nasal tip, thin upper lip, and triangular face in individual 5 from the study of Nizon et al. were also detected in this child. This child was affected by chronic constipation, which was also reported in individuals from the study of Nizon et al. The brain MRI of individuals 4 and 5 from the study of Nizon et al. detected agenesis or hypoplasia of the corpus callosum (12). Nevertheless, in this case, brain MRI did not reveal obvious structural abnormalities in the corpus callosum. In line with the findings of previous studies, not every individual with single-gene disorders, such as Sotos syndrome and Rubinstein–Taybi syndrome, displayed agenesis of the corpus callosum (23). This phenomenon indicates that some genes only partially contribute to callosal formation, and agenesis of the corpus callosum may be caused by polygenic and complex interactions (23). An enlarged PVS was detected in the right temporal lobe. Previous studies on carriers of *PTEN* mutations have also revealed enlarged PVS in individuals with autistic features (24, 25). However, a recent study showed that FMR1 premutation carriers with fragile X-associated tremor/ataxia syndrome exhibited higher ratings of PVS in the basal ganglia than controls (26). Thus, these findings indicate that an enlarged PVS is not a specific imaging sign for autistic individuals carrying LoF variants (27).

MED12L, as a part of the Mediator complex involved in transcriptional coactivation, is predicted to be a subunit of the kinase module with MED12, MED13, MED13L, CDK8, CDK19, and Cyclin C. Previous studies on genetics and animal models have revealed that dysfunction of subunits of the kinase module may be involved in the development of ASD. Two sequencing studies with large-scale cohorts found that the *de novo* mutation rate of *MED13* and *MED13L* in individuals with autism spectrum disorders was higher than that in controls or the non-neuro subset from gnomAD exomes (6, 28). Additionally, studies of animal models have found that dysfunction of subunit genes consisting of the kinase module exerts adverse effects on neuronal and brain development. As two subunits of the kinase module, the loss of function of Cdk8 and Med13 was verified to disrupt axon navigation in *C. elegans* neurons (29). In a zebrafish model, disruption of the Mediator subunit Med12 resulted in deficits in forebrain serotonergic (5HT) neurons and hindbrain 5HT neurons (30). In a mouse model, *MED13L* disease-associated variants disrupted dendritic growth and the formation of mature dendritic spines (31). Therefore, as a subunit of the kinase module, the role of MED12L in the development of autism needs to be elucidated in future studies.

Moreover, multiple studies in animal models have indicated that several high-confidence ASD genes, including CHD8, ARID1B, ADNP, and TBR1, regulate Wnt/β-catenin-dependent transcriptional activity in multiple neuronal cell types (32). Previous studies in mice and rat models of autism have revealed that dysregulation of Wnt/β-catenin signaling may increase susceptibility to autism-like phenotypes (33, 34). According to the results from the InterPro database (https://www.ebi.ac.uk/interpro/protein/UniProt/Q86YW9/), MED12L has eukaryotic Mediator 12 subunit domain (Med12-LCEWAV) and eukaryotic Mediator 12 catenin-binding domain (Med12-PQL). A previous study *in vitro* and *in vivo* found that Med12-PQL bound directly to isolated Med12 and intact Mediator and was involved in the regulation of Wnt/β-catenin signaling (35). Therefore, these findings indicate that dysfunction of MED12L might be involved in the development of ASD by disrupting the Wnt/β-catenin signaling pathway and that *MED12L* might be a potential candidate gene for ASD.

This case report has several limitations. First, the inference of the association between this truncating mutation and severe autistic features was based on a single proband. Due to the rarity of LoF variants of *MED12L*, replication in other cases with comparable phenotypic features is required to validate this observation. Second, functional studies of this truncating mutation have not been performed. The deleterious effect of this truncating mutation should be demonstrated through *in vitro* and *in vivo* experiments. The relationship between this truncating mutation and dysregulation of Wnt/β-catenin signaling in neuronal development remains to be elucidated. Third, detailed information on early interventions, educational support, and rehabilitation services was not systematically described, precluding evaluation of the potential influence of truncating mutations on intervention efficacy and developmental outcomes. Finally, although structural MRI revealed no hypoplasia or atrophy of the corpus callosum, the absence of functional MRI failed to assess interhemispheric functional connectivity.

## Conclusion

This case report suggests that *de novo* truncating mutations resulting in haploinsufficiency of *MED12L* may be associated with autism. However, several problems need to be addressed. The effects of this novel *de novo* truncating mutation in *MED12L* on clinical manifestations warrant further functional studies in animal models to elucidate its relationship with ASD. Moreover, the relationship between *MED12L* and corpus callosum function should be further demonstrated through functional MRI studies.

## Data Availability

The whole exome sequencing data and psychological assessments for this article are not publicly available due to concerns regarding individual anonymity. Requests for access to these data should be directed to the corresponding authors.
