# Supplementary material for: Case Report: A novel de novo heterozygous truncating mutation in MED12L identified in a Chinese autistic boy
Source: Front Psychiatry. 2026 Jul 13;17:1824663. doi: 10.3389/fpsyt.2026.1824663 (PMC13403109; doi:10.3389/fpsyt.2026.1824663)
Supplement: Supplementary file 1 [file Supplementaryfile1.docx]

Figure S1. A timeline with relevant data from the development assessment and detailed information about the intervention


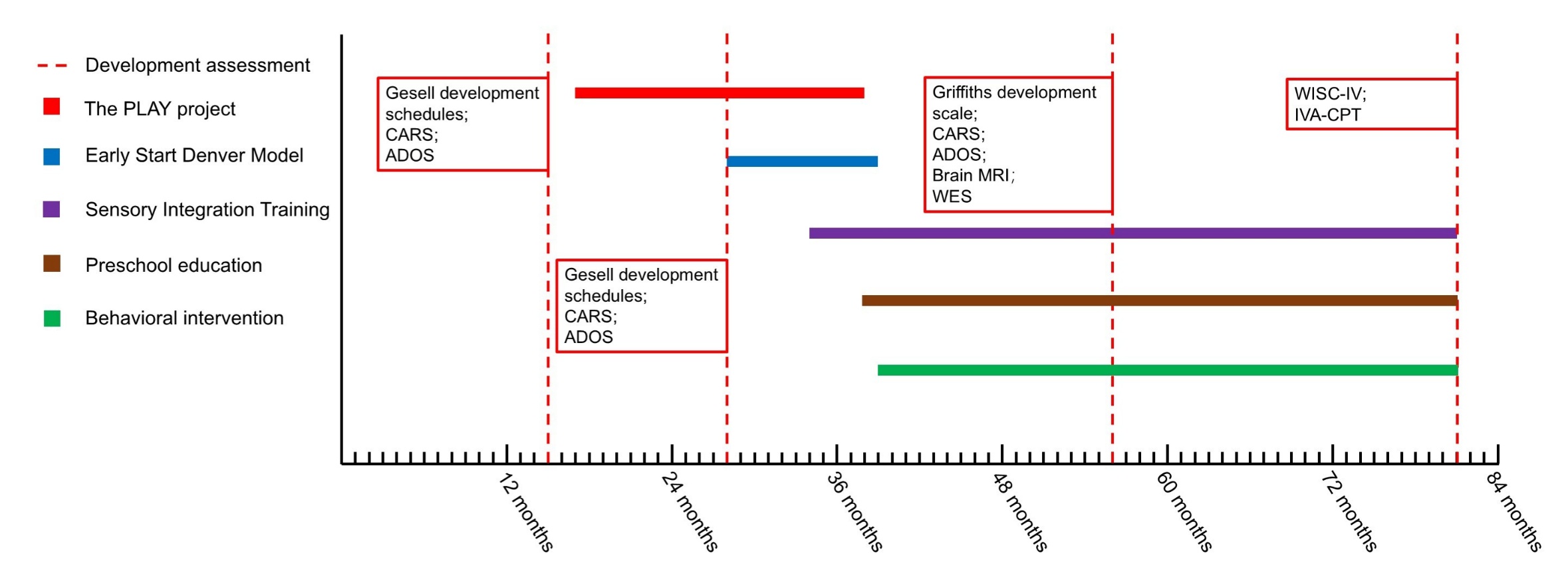


Abbreviations: PLAY, the Play and Language for Autistic Youngsters; CARS, Childhood Autism Rating Scale; ADOS, Autism Diagnostic Observation Schedule; MRI, Magnetic Resonance Imaging; WES, Whole-exome Sequencing; WISC-IV, Wechsler Intelligence Scale for Children–Fourth Edition; IVA-CPT, Integrated Visual and Auditory Continuous Performance Test
